# Retraction: Unique and outstanding catalytic behavior of a novel MOF@COF composite as an emerging and powerful catalyst in the preparation of 2,3-dihydroquinazolin-4(1*H*)-one derivatives

**DOI:** 10.1039/d4na90124j

**Published:** 2024-11-29

**Authors:** Mohammad Ali Ghasemzadeh, Boshra Mirhosseini-Eshkevari

**Affiliations:** a Department of Chemistry, Qom Branch, Islamic Azad University Post Box: 37491-13191 Qom Iran qasemzade.a@gmail.com ma.ghasemzadeh@iau.ac.ir

## Abstract

Retraction of ‘Unique and outstanding catalytic behavior of a novel MOF@COF composite as an emerging and powerful catalyst in the preparation of 2,3-dihydroquinazolin-4(1*H*)-one derivatives’ by Mohammad Ali Ghasemzadeh *et al.*, *Nanoscale Adv.*, 2023, **5**, 7031–7041, https://10.1039/D3NA00805C.

The Royal Society of Chemistry hereby wholly retracts this *Nanoscale Advances* article due to concerns with the reliability of the data.

The XRD patterns in Fig. 2 have repeating segments between the traces, the SEM image of the reused catalyst in Fig. 10a is the same as other SEM images found in other publications, describing different materials.

In the ESI, the ^13^C NMR spectra of 4m (Fig. S3) and 6g (Fig. S6) have repeating patterns and the XRD patterns in Fig. S9 and S10 are identical.

Given the significance of these concerns, the findings presented in this paper are no longer reliable.

The authors were informed about the retraction of the article. Mohammad Ali Ghasemzadeh agreed with the decision, Boshra Mirhosseini-Eshkevari has not responded.

Signed: Mohammad Ali Ghasemzadeh

Date: 14th November 2024

Retraction endorsed by Jeremy Allen, Executive Editor, *Nanoscale Advances*

